# The gravitational force of mental health services: distance decay effects in a rural Swiss service area

**DOI:** 10.1186/s12913-018-2888-1

**Published:** 2018-02-05

**Authors:** Niklaus Stulz, Eva-Maria Pichler, Wolfram Kawohl, Urs Hepp

**Affiliations:** 1 Psychiatric Services Aargau, P.O. Box 432, 5201 Brugg, Switzerland; 2 Integrated Psychiatric Services Winterthur – Zurcher Unterland, P.O. Box 144, 8408 Winterthur, Switzerland

**Keywords:** Mental health services, Distance, Geography

## Abstract

**Background:**

Previous research suggested a distance decay effect in health services systems, with people living closer to service facilities being more likely to use them.

**Method:**

In this ecological cross sectional study, we conducted spatial and statistical analyses in a Swiss mental health services system being legally bound to provide primary mental health care to approximately 620,000 inhabitants. We examined a cohort of all patients who were over 18 years old and who were treated in the mental health services system between January and December 2011.

**Results:**

There were 5574 treatment cases during the 12-month period, 2161 inpatient cases and 3413 outpatient cases. Travel time by public transportation between patients’ residence and the closest mental health service facility negatively predicted the utilization of outpatient services for all mental disorders, even after controlling for variability in ecological (e.g. socioeconomic) characteristics of the communities in the service provision area. For utilization of inpatient wards no geographical distance decay effect was observed, except for organic mental disorders.

**Conclusions:**

Based on these findings, outpatient clinics should be most effectively located decentralized and in the largest communities to meet the needs of the population as close as possible to where people live and to avoid remote areas being insufficiently supplied with mental health care. For mental hospitals and inpatient services decentralized location seems to be less important.

## Background

In an ideal health care system all people should have equal access to medical care [1]. Accessibility of medical services depends on both non-spatial factors (such as economic, cultural, and social issues or factors related to the organization of the health care system) and on spatial factors (such as geographical distance) [2–4]. Back in 1866, Jarvis [5] identified an inverse relationship between hospitalization rates and the distance between the patients’ residences and the location of the hospital, with people living closer to hospitals being more likely to use them. To date, such ‘distance decay effect’ has been replicated with remarkable persistence by numerous studies that suggest a universal pattern of reduced service utilization with increasing spatial and time-related distance between peoples’ residences and somatic [2, 6–10] and psychiatric [1, 11–22] service sites.

Importantly, distance decay effects were not only found for mental hospitals but also for outpatient clinics. Bürgy and Häfner-Ranabauer [18], for example, explored the relationship between the utilization of a psychiatric emergency service in Mannheim, Germany, and the accessibility of that outpatient service for the inhabitants in its catchment area. When using the spatial distance (air distance) and the time-related distance (travel time required by public transportation) between patients’ residence and the location of the emergency service as a proxy of its geographical accessibility, they found an association between increasing home-to-service site distance and reduced contacts with the emergency service. Beyond geographical proximity, less favorable ecological conditions of a district (e.g., higher population density, worse housing conditions, or higher proportion of foreigners) and certain diagnoses (e.g. schizophrenia and substance use disorders) did likewise predict increased outpatient service utilization in that German study. Interestingly, however, there was no interaction effect between distance and diagnoses on service utilization; that is, service utilization decreased with increasing home-to-service site distance for all mental disorders to the same degree with no differences between diagnoses. The findings from that German study are highly interesting but they might not generalize to more comprehensive psychiatric outpatient services which also provide non-emergency care in less urban environments since the study was restricted to the utilization of an emergency facility during out of office hours in an urban area.

In a more recent study, Zulian et al. [1] assessed the influenced of distance and of clinical and socioeconomic patient characteristics on the utilization of community-based mental health services in Verona, Italy. Spatial and statistical analyses unfolded a distance decay effect with different trends for the three types of community-based mental health services under examination: the strongest negative correlation between distance and the number of patients per inhabitant in a specific area (caseload) was observed for outpatient clinics, followed by community mental health centers offering day care and rehabilitation. For acute inpatient wards the correlation was the least pronounced but still statistically significant. When controlling for socioeconomic predictors of service utilization (such as age, gender, marital status, or occupational status), the aforementioned distance decay effects were even stronger. That is, the influence of distance on service utilization was underestimated in this service provision area if the influence of unequally distributed socioeconomic variables on service utilization was omitted. Despite examining the impact of diagnosis – and of other characteristics (such as age, gender, etc) – on service utilization, Zulian et al. desisted from analyzing whether distance decay effects vary depending on diagnoses. With respect to service planning, however, it may be important to know which patients “suffer” the most from a distance decay effect. Zulian et al. furthermore used the travel time by car as a proxy of distance. This might be a little precise indicator of spatial accessibility for those patients who do not have access to a car or who are not able to drive a car (which might particularly often apply to mentally ill people).

To the best of our knowledge, no past study on distance decay effects in mental health care settings unified the following characteristics: (a) spatial accessibility was assessed in terms of travelling time by public transportation (which is available to everybody); (b) inpatient and outpatient services were both considered allowing for comparisons between settings; (c) the sample included regular inpatient and outpatient services (i.e., it was not restricted to psychiatric emergency services); and (d) distance effects were examined depending on diagnostic groups. We therefore aimed at answering the following research questions based on data from one of the largest service provision areas in Switzerland:

1. Does distance (i.e., travel time by public transportation) between patients’ homes and mental health service facilities affect the utilization of inpatient and outpatient services, respectively?
2. Does the relationship between distance and service utilization depend on the primary diagnosis of the patients?

## Methods

### Setting

**Fig. 1:**
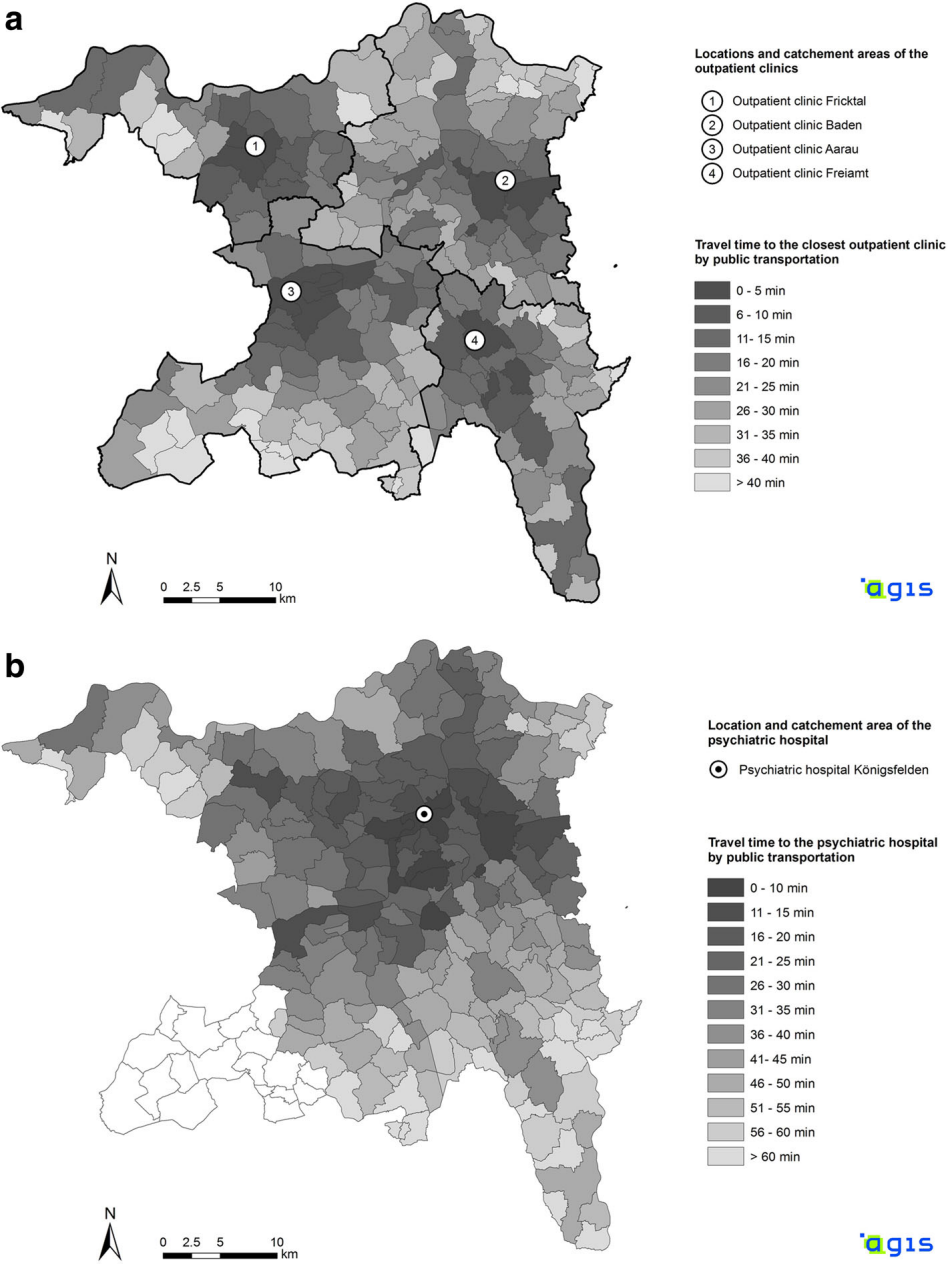
**a** and **b** Travel time by public transportation from the 219 communities to: (**a**) the 4 outpatient clinics, and (**b**) the mental hospital in the Canton of Aargau. Source: Data of the Canton of Aargau

This ecological cross sectional study on distance decay effects in a mental health services system was conducted in the Canton of Aargau. The Canton of Aargau is a federal state in the northern part of Switzerland with approximately 620,000 inhabitants (census data 2011) who are living in 219 political municipalities [23]. The Psychiatric Services Aargau (PDAG) are responsible for the vast majority of institutional and public mental health care in the Canton of Aargau. The PDAG run one mental hospital (320 beds), which is located in the center of the Canton (Fig. 1b). This mental hospital is responsible for inpatient care of all inhabitants of the Canton of Aargau and, in 2011, it delivered approximately 85% of all inpatient treatments provided by public mental hospitals in the federal state [24, 25]. In addition to inpatient services, the PDAG operate 4 community-based outpatient clinics, each serving a specific catchment area and each being located in a larger community of the respective catchment area (Fig. 1a).

### Sample

This study examined a cohort of all patients who were over 18 years old and who were treated in the PDAG between January 1, 2011 and December 31, 2011. The primary study unit was a treatment case in the mental hospital of the PDAG or in one of its outpatient clinics, respectively. Furthermore, the number of outpatient visits and the number of inpatient days during this one-year study period were considered as indicators of treatment intensity. Inpatient treatments in the forensic wards of the mental hospital were excluded from our analyses as these wards accommodate a highly specific type of involuntary patients for which distance decay effects may look considerably different. We furthermore excluded the inhabitants of the 18 municipalities of the region of Zofingen from our analyses on the utilization of inpatient services (Fig. 1b). The inhabitants of that region in the South-Western part of the Canton of Aargau were very frequently treated in the mental hospital of the neighboring Canton of Lucerne which is located right at the boarder of the Canton of Aargau and hence is much closer than the hospital of the PDAG for the inhabitants in the region Zofingen.

#### Utilization of psychiatric services: Caseload and treatment intensity

The number of inpatient treatment cases and the number of outpatient treatment cases per inhabitant in a particular municipality *i*, the *caseload (CL*_*i*_*)*, was calculated as follows:

$$ {\mathrm{C}\mathrm{L}}_{\mathrm{i}}={\mathrm{C}}_{\mathrm{i}}/{\mathrm{P}}_{\mathrm{i}} $$

where *C*_*i*_ represents the number of treatment cases in municipality *i* during the 1 year study period (data were derived from the medical database of the PDAG), and *P*_*i*_ represents the population of community *i* (data were derived from census data 2011) [23]. These caseloads were subsequently standardized for both settings (inpatient and outpatient) separately by dividing them by their respective maximum value. This enabled for comparisons of the degree of the distance decay effects between the two settings (see below).

*Treatment intensity (TI*_*i*_*)*, i.e., the number of outpatient visits and the number of inpatient days per inhabitant in a community *i*, was calculated as follows:

$$ {\mathrm{T}\mathrm{I}}_{\mathrm{i}}={\mathrm{T}}_{\mathrm{i}}/{\mathrm{P}}_{\mathrm{i}} $$

where *T*_*i*_ represents the number of outpatient visits or the number of inpatient days, respectively, in municipality *i* during the 1 year study period, and *P*_*i*_ represents the population of the community *i*.

#### Spatial and statistical analyses

There is an excellent public transportation network in Switzerland in general and in the Canton of Aargau in particular. The Swiss government makes every effort to convince people to use railways and buses instead of private cars. Because public transportation is accessible to (almost) everybody, for this study we considered the travel time between peoples’ residences (i.e., the main public transportation station in every community) and the service facilities (mental hospital or outpatient clinic) by public transportation to be the most valid available indicator of the geographical accessibility of the treatment facilities of the PDAG. Using the nationwide online timetable for public transportation provided by the Swiss Public Railways company (www.sbb.ch), we considered the first 4 connections after 10 am on weekdays. The fastest of these four connections was chosen as indicator of the distance between patients’ homes and the closest outpatient clinic (Fig. 1a) or the mental hospital (Fig. 1b), respectively. Note that due to express trains between larger communities the longest geographical distances were not necessarily associated with the longest travel times.

Statistical analyses were performed in two steps: First, we used multiple regression analysis to see whether distance (i.e., travel time by public transportation) to the closest service facility was related to service utilization at that treatment site when controlling for potentially confounding ecological characteristics of the communities. Subsequently, we pooled the communities based on similar distance ranges to the closest service facility in order to examine the extent (effect size) and the shape of the distance decay effect within diagnostic subgroups.

For our primary analyses to examine the association of distance with the utilization of psychiatric in- and outpatient services, respectively, we used multiple regression analyses. Continuous dependent variables were either the caseload (i.e., the number of in- or outpatient cases per inhabitant in a community) or the treatment intensity (i.e., the number of outpatient visits or inpatients days per inhabitant in a community). In addition to distance (travel time), we entered all publicly available ecological characteristics of the communities (e.g., tax amount per inhabitant, cf. [23]) as independent variables into the multiple regression model in order to control for potentially confounding variables. Confidence intervals of the growth parameters were estimated using bootstrapping methods (*k* = 5000 samples) to account for violations of the normal distribution. (Note that the Poisson regression models, which were used in some previous studies on distance decay effects [1], require count data (i.e., integer values equal or greater than zero) which made Poisson regression inappropriate for the continuous ratio scores in our dependent variables.)

In a series of subsequent analyses we examined distance decay effects within the most prevalent diagnostic subgroups (ICD-10: F0, F1, F2, etc.) separately. In order to render reliable estimates of the distance decay effects within specific diagnostic subgroups, we (a) restricted our analyses to those primary diagnoses which were present in *n* ≥ 200 cases, and we (b) categorized municipalities with similar traveling times to the closest treatment facility into distance range categories around the 4 outpatient clinics and the mental hospital, respectively. This categorization was based on 5 min intervals (e.g., all municipalities with a traveling time of 10-15 min to the closest treatment facility were merged into one distance range category). The corresponding aggregation of caseloads across municipalities within the same distance range should help to correct for potential outliers in very small municipalities and hence to provide more reliable estimates of the caseload. Finally, if there were less than 10 municipalities within a specific distance range, we merged the neighboring distance range(s) until the resulting distance range included at least 10 municipalities. This again was intended to provide more reliable estimates of the caseloads within diagnostic subgroups since the caseload in each distance range relied on at least 10 observations (municipalities). Bivariate associations between the average travel time per distance range category and the corresponding caseload per distance range category were examined using non-parametric Spearman rank correlations in order to account for the rank ordered distance range categories within diagnostic subgroups.

All analyses were performed with SPSS 18 [26].

#### Ethics

The study used de-identified linked administrative data. The responsible ethics committee Northwest/Central Switzerland declared that there is no need for approval, as the study does not fall under the Swiss National Law on Human Research (UBE-2017-00281). However, the ethics committee confirmed agreement with general ethical principles and declared the unproblematic nature of the study from an ethical point of view (cf. article 51, paragraph 2 of the Swiss National Law on Human Research).

## Results

Table 1 shows the characteristics of the 5574 treatment cases in the PDAG in 2011. There were 2161 inpatient cases with a total of 75,430 inpatient days (*M* = 34.9 inpatient days per case; *SD* = 44.8), and 3413 outpatient cases with a total of 16,855 contacts to the outpatient clinics (*M* = 4.9 contacts per case; *SD* = 6.1).

**Table 1:** Characteristics of the treatment cases by type of setting

	Outpatient clinics (*n* = 3413 cases)	Inpatient wards (*n* = 2161 cases)	Total (*n* = 5574 cases)
Gender: Male, *n* (%)	1661 (48.7)	1116 (51.6)	2777 (49.8)
Age, *M* (*SD*) [Range]	40.7 (14.5) [18-90]	49.6 (19.5) [18-97]	44.1 (17.1) [18-97]
Nationality, *n* (%)^a^			
Swiss	2733 (83.6)	1954 (91.4)	4687 (86.7)
Other	538 (16.4)	184 (8.6)	722 (13.4)
Diagnostic groups (ICD-10), *n* (%)			
F0 (Organic, including symptomatic, mental disorders)	51 (1.5)	331 (15.3)	382 (6.9)
F1 (Mental and behavioral disorders due to psychoactive substance use)	170 (5.0)	485 (22.4)	655 (11.8)
F2 (Schizophrenia, schizotypal and delusional disorders)	548 (16.1)	413 (19.1)	961 (17.2)
F3 (Mood [affective] disorders)	1105 (32.4)	484 (22.4)	1589 (28.5)
F4 (Neurotic, stress-related and somatoform disorders)	956 (28.0)	253 (11.7)	1′209 (21.7)
F6 (Disorders of adult personality and behavior)	145 (4.2)	122 (5.6)	267 (4.8)
F9 (Behavioral and emotional disorders with onset usually occurring in childhood and adolescence)	135 (4.0)	10 (0.5)	145 (2.6)
Z (Factors influencing health status and contact with health services)	126 (3.7)	8 (0.4)	134 (2.4)
Other	105 (3.1)	33 (1.5)	138 (2.5)
Unknown	72 (2.1)	22 (1.0)	94 (1.7)

^a^Data of *n* = 142 outpatient cases and of *n* = 23 inpatient cases were missing

### Distance decay effects in outpatient services

**Fig. 2:**
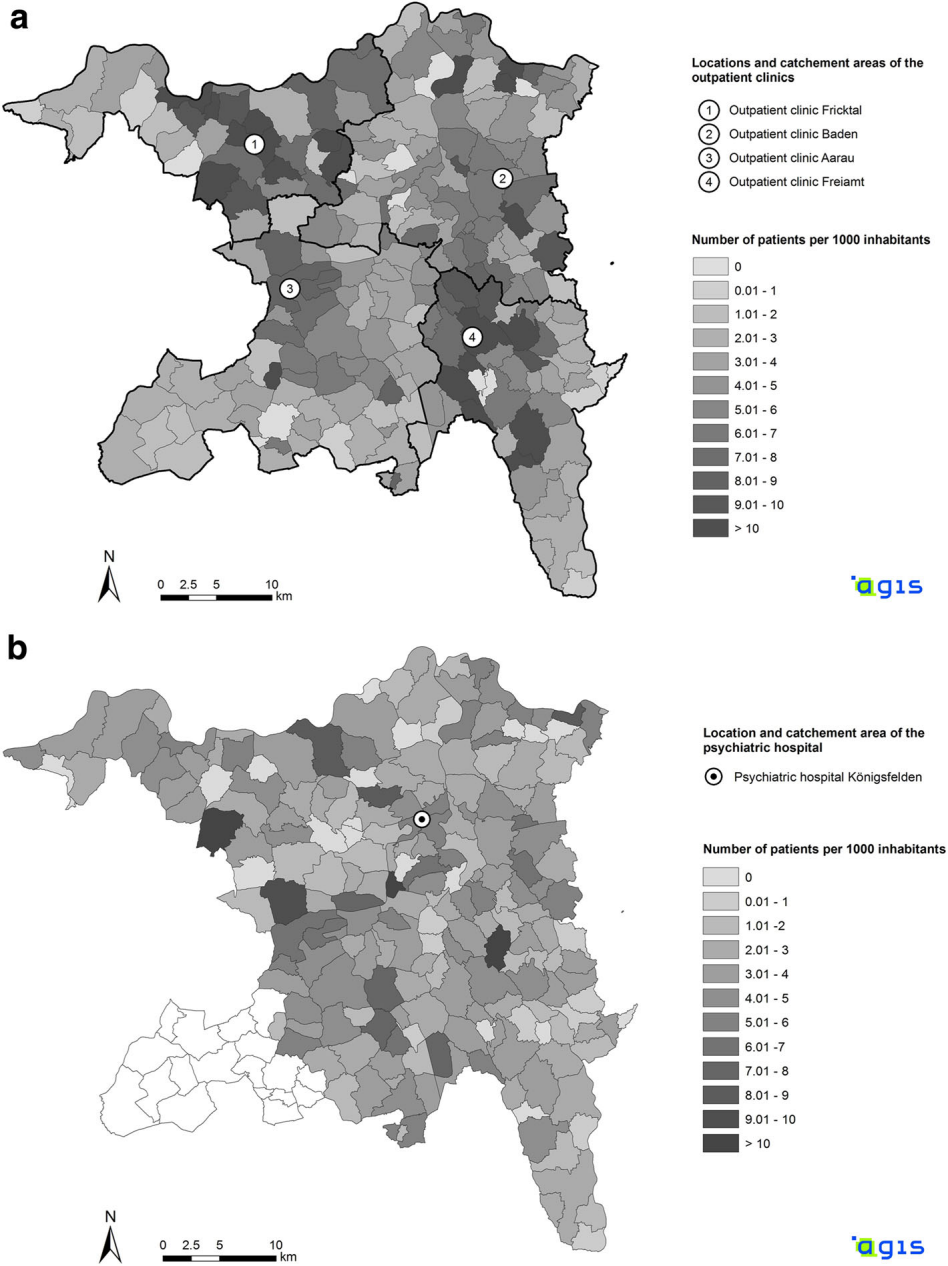
**a** and **b** Caseloads (treatment cases per 1000 inhabitants) in the municipalities of the Canton of Aargau: (**a**) *n* = 3413 outpatient cases in the 4 outpatient clinics, and (**b**) *n* = 2161 inpatient cases on the wards of the mental hospital. Source: Data of the Canton of Aargau

There was a distance decay effect for the utilization of outpatient clinics (Table 2 and Fig. 2a). Travel time by public transportation between the communities and the outpatient clinics negatively predicted both the number of outpatient cases per inhabitant (*B* = − 0.108; 95% CI = − 0.154, − 0.062; *p* < .001) and the number of outpatient visits per inhabitant (*B* = − 0.816; 95% CI = − 1.214, − 0.453; *p* < = .001) when controlling for all available ecological characteristics of the communities in multiple linear regression models (Table 2). (Multiple linear regression models were chosen because they had outperformed exponential regression models in preliminary analyses with non-stratified distance data (outpatient cases: *R*^*2*^ = 0.174 vs. 0.067, outpatient visits: *R*^*2*^ = 0.125 vs. 0.069)).

**Table 2:** Multiple regression analyses: Prediction of outpatient service utilization by distance (minutes in public transportation between patient’s homes and outpatient clinics) and by available ecological variables of the communities

	Outpatient cases per capita				Outpatient visits per capita			
Characteristic of the community	*B* (95% CI)	*SE*	*β*	*p*	*B* (95% CI)	*SE*	*β*	*p*
Constant	15.701 (−7.453, 37.059)	11.506		.171	39.852 (− 177.144, 242.107)	106.193		.711
Distance (travel time in minutes)	−0.108 (−0.154, − 0.062)	0.023	− 0.372	<.001	− 0.816 (−1.214, − 0.453)	0.196	− 0.367	.001
Age (mean in years)	− 0.110 (− 0.352, 0.152)	0.127	− 0.056	.379	0.541 (− 1.277, 2.551)	0.970	0.036	.579
Female (%)	− 7.089 (−50.236, 37.843)	22.470	− 0.024	.746	−10.065 (− 416.011, 391.978)	207.621	− 0.004	.959
Immigrants (%)	5.332 (0.390, 10.363)	2.518	0.140	.039	9.794 (−26.440, 44.041)	17.842	0.034	.589
Taxes (mean per inhabitant in Swiss Francs)	0.000 (−0.001, 0.000)	0.000	−0.082	.213	−.006 (−0.011, − 0.001)	.003	−.0128	.025

Outpatient cases per capita: *R*^*2*^ = .206, *p* < .001. Outpatient visits per capita: *R*^*2*^ = .142, *p* < .001

*B* = Regression weight

95% confidence intervals (CI) were estimated with bootstrap methods (based on *k* = 5000 samples)

*SE* = Standard error

*β* = Standardized regression weight

**Fig. 3:**
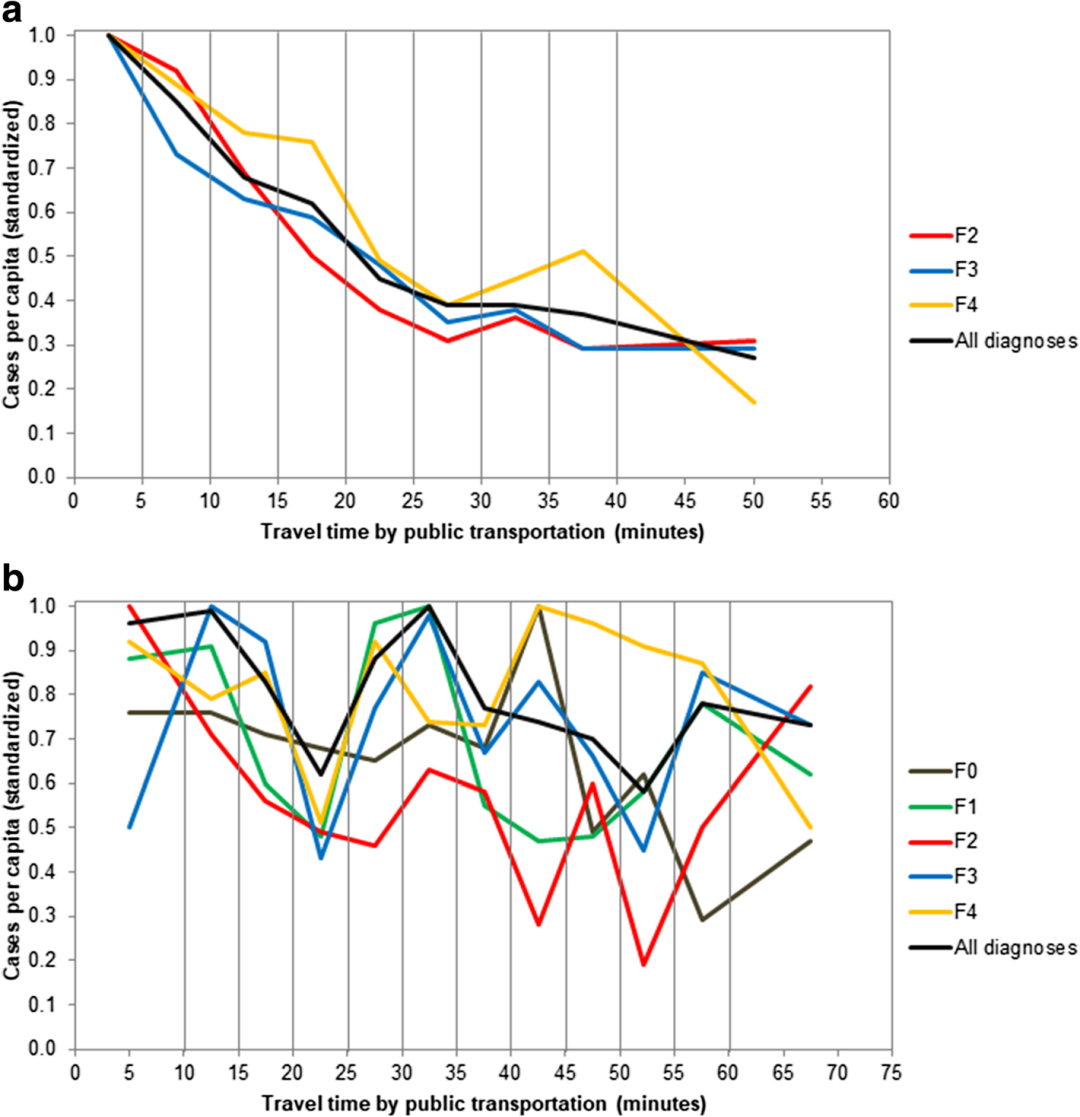
**a** and **b** Standardized distance effects of stratified travel times by public transportation on caseloads (treatment cases per inhabitant): (**a**) outpatient clinics, and (**b**) inpatient wards. Note: F0 = Organic, including symptomatic, mental disorders; F1 = Mental and behavioral disorders due to psychoactive substance use; F2 = Schizophrenia, schizotypal and delusional disorders; F3 = (Mood [affective] disorders); F4 = Neurotic, stress-related and somatoform disorders

For subsequent analyses to examine the degree of distance decay effects and their shape within diagnostic subgroups, the *n* = 219 communities were categorized into 9 distance ranges (based on 5 min intervals). Across all primary diagnoses, the standardized caseload (i.e., the standardized number of cases per inhabitant) decreased monotonically with increasing distance between patients’ residence and outpatient facilities (*r*_*s*_ = −.999; *n* = 9; *p* < .001). In municipalities being located more than 20 min away from the closest outpatient clinic, the caseload was reduced by more than 50% (Fig. 3a). This trend went on up to 60 min (standardized caseload: 27%), though it began to level out at a distance of 30 min away from the closest outpatient clinic (standardized caseload: 39%). Regarding distance decay effects within diagnostic subgroups, the number of patients who were enrolled in outpatient treatment (i.e., the caseload) decreased with increasing distance to outpatient clinics in all diagnostic subgroups with at least *n* = 200 cases: F2 (*r*_*s*_ = −.917; *n* = 9; *p* = .001), F3 (*r*_*s*_ = −.967; *n* = 9; *p* < .001), and F4 (*r*_*s*_ = −.883; *n* = 9; *p* = .002). (Note that *n* = 72 (2.1%) outpatient cases were excluded from these analyses due to missing data on the primary diagnosis.) Differences between the distance decay effects of the primary diagnoses were only marginal (Fig. 3a).

### Distance decay effects in inpatient services

In contrast to outpatient services, the home-to-hospital distance did neither predict the number of inpatient cases per inhabitant (*B* = − 0.014; 95% CI = − 0.036, 0.008; *p* = .214) nor the number of inpatient days per capita (*B* = − 0.748; 95% CI = − 1.618, 0.108; *p* = .120) when controlling for the available ecological characteristics of the communities in multiple linear regression models (Table 3 and Fig. 2b). However, a higher proportion of immigrants in communities was associated with higher inpatient service utilization in the respective communities (Table 3). (Multiple linear regression models were again given preference because they had outperformed exponential regression models in preliminary analyses with non-stratified distance data (inpatient cases: *R*^*2*^ = 0.039 vs. 0.013, inpatient days: *R*^*2*^ = 0.029 vs. 0.012)).

**Table 3:** Multiple regression analyses: Prediction of inpatient service utilization by distance (minutes in public transportation between patient’s homes and the mental hospital) and by available ecological variables of the communities

	Inpatient cases per capita				Inpatient days per capita			
Characteristic of the community	*B* (95% CI)	*SE*	*β*	*p*	*B* (95% CI)	*SE*	*β*	*p*
Constant	−5.515 (−18.375, 7.396)	6.493		.396	− 130.031 (− 608.273, 453.067)	271.087		.694
Distance (travel time in minutes)	−0.014 (− 0.036, 0.008)	0.011	− 0.101	.214	− 0.748 (−1.618, 0.108)	0.440	−0.120	.102
Age (mean in years)	0.018 (−0.131, 0.184)	0.081	0.014	.829	−1.342 (−9.410, 6.165)	3.936	−0.023	.731
Female (%)	14.807 (−11.975, 41.981)	13.505	0.077	.274	518.290 (−620.705, 1562.056)	554.487	0.059	.348
Immigrants (%)	7.394 (3.995, 10.828)	1.735	0.291	<.001	196.137 (52.491, 338.146)	72.500	0.169	.012
Taxes (mean per inhabitant in Swiss Francs)	−0.000 (−0.001, 0.000)	0.000	−0.015	.820	0.001 (−0.016, 0.019)	0.009	0.003	.949

Inpatient cases per capita: *R*^*2*^ = .118, *p* < .001. Inpatient days per capita: *R*^*2*^ = .059, *p* = .036

*B* = Regression weight

95% confidence intervals (CI) were estimated with bootstrap methods (based on *k* = 5000 samples)

*SE* = Standard error

*β* = Standardized regression weight

In line with the above findings, across all primary diagnoses, there was no statistically significant distance decay effect if the communities were categorized into 12 similar distance ranges based on 5 min intervals (*r*_*s*_ = −.573, *n* = 12, *p* = .051). As can be seen in Fig. 3b, the caseload varied considerably between the distance ranges. After being clearly reduced at a distance of 20-25 min away from the mental hospital (standardized caseload: 62%), the number of inpatients per inhabitant increased again and reached a second peak at a travel time of 30-35 min to the hospital (standardized caseload: 100%).

Regarding the specific diagnostic subgroups with at least *n* = 200 cases, the only statistically significant distance decay effect was evident for F0 diagnoses; that is, in patients with organic mental disorders the caseload decreased with increasing distance between their residence and the mental hospital (*r*_*s*_ = −.713; *n* = 12; *p* = .009). In all remaining diagnostic subgroups with at least n = 200 cases there was no indication for a distance decay effect: F1 (*r*_*s*_ = −.280; *n* = 12; *p* = .379); F2 (*r*_*s*_ = −.266; *n* = 12; *p* = .404); F3 (*r*_*s*_ = −.133; *n* = 12; *p* = .681); and F4 (*r*_*s*_ = −.021; *n* = 12; *p* = .948). (Note that *n* = 22 (1.0%) inpatient cases were excluded from these analyses due to missing data on the primary diagnosis.)

## Discussion

Research on spatial factors affecting the accessibility of mental health services has repeatedly demonstrated a pattern of reduced service utilization with increasing spatial or time-related distance between peoples’ residences and service facilities [1, 11–21]. Our results corroborate previous findings of such a distance decay effect in the outpatient setting [1, 16–20, 22]. Importantly, we used the travel time by public transportation, which is available to (almost) everybody, as proxy for the distance between patients’ residence and the outpatient clinic. Furthermore, we controlled for the influence of available socioeconomic characteristics of the communities (e.g., the average tax amount per inhabitant) on service utilization. While a distance decay effect was evident in our outpatient setting irrespective of the type of mental disorder (i.e., for all of the most prevalent ICD-10 primary diagnoses), no such distance decay effect was found in our inpatient setting, except for patients with organic mental disorders (ICD-10: F0). A possible explanation would be that F0 diagnoses such as dementia occurred almost exclusively in elderly patients who are among the least mobile members of the society.

Differences in distance decay effects between the inpatient and outpatient treatment settings may be expected given that outpatients have to travel the home-to-service facility distance for every single visit in an outpatient clinic, whereas inpatients have to cover the home-to-hospital distance only twice (when entering the hospital and after discharge). In addition, the more severe and acute clinical condition of inpatients, who are often in need of emergency and/or involuntary admissions, may likewise mitigate the influence of distance on the utilization of inpatient wards [27]. Even though the lack of a global distance decay effect in our mental hospital contrasts with some previous findings [5, 12–15], other earlier studies did also not find a clear-cut association between the home-to-hospital distance and the probability of being hospitalized [28, 29], or they at least reported less pronounced distance decay effects for inpatient services [1]. The complete lack of a distance decay effect except for organic mental disorders in our data might be explained by the rural location of our hospital, with some larger cities in more remote areas of the hospital. As a result, in our service provision area any distance decay effect might have been outbalanced by higher caseloads in these remote cities. It is well known that urban areas typically have higher rates of mental disorders than rural areas [30–35]. In fact, the first peak of the inpatient caseload in our service area, which occurred at a distance range of 10-15 min away from the hospital (Fig. 3b), may be explained by the high caseloads in the cities of Aarau and Wettingen (which are the most populous communities in our service area). Thus, in previous studies where mental hospitals were located close to the most populated urban centers, the finding of a distance decay effect might simply have reflected some decay in the prevalence of mental disorders when moving from urban to rural areas. Such “typical ecological distribution” or gradient of mental disorders is sometimes explained by the less favorable social and ecological conditions in urban areas [31]. In line with this, our analyses, which included all available ecological characteristics of the communities as potential confounders, revealed that the proportion of immigrants – which is typically higher in urban communities and which may reflect less favorable social and ecological conditions – was predictive of inpatient service utilization. Such association between a high concentration of immigrants from foreign countries in a geographical area and a more intense utilization of inpatient [32] and of outpatient [18] services in that geographical area has already been reported by previous studies. It is important to note, however, that being an immigrant per se is not a risk factor for mental health service utilization. Immigrants do not more often use mental health services themselves. On the contrary, in our service provision system they were even underrepresented among inpatients (they accounted for 8.6% of the inpatient cases while they represented 22.8% of the Swiss population in 2011 [23]). Thus, rather than immigrant status being an individual risk factor itself, it is the concentration of immigrants in a community that indicates a higher risk for inpatient utilization of inhabitants, probably because the concentration of immigrants represents less favorable social structure in that respective community which in turn increases the risk for inpatient utilization [36].

In summary, our findings and their comparison with results from previous studies support the notion that the distance decay relationship in mental health services is not a simple and consistent one but rather results from a complex interaction between geographical proximity to services, socioeconomic conditions in local communities, the organization of mental health services, and the transportation infrastructure [2, 3].

With regard to practical implications, some recommendations can be derived from our findings for the most effective location of mental health services. The distance decay effect in our outpatient clinics, which seemed to occur irrespective of the type of mental disorder, denotes the importance of decentralized outpatient clinics to meet the needs of the population as close as possible to where people live and to avoid people in remote areas being insufficiently supplied with mental health care. At a distance of 25 min traveling time by public transportation to the closest outpatient clinic, the proportion of inhabitants using our outpatient services (the caseload) was already reduced by more than 50%. Furthermore, the spatial accessibility of our outpatient clinics did not only affect the caseload (i.e., the proportion of inhabitants receiving any outpatient treatment at all) but it also affected the number of outpatient sessions that the patients received [37]. This requires particular attention given that frequency and continuity of care are known to be important components for the effectiveness of outpatient treatment [38]. If decentralized location of outpatient clinics is not possible for any reason, the provision of transportation services as a part of mental health care programs could be a promising way to enable equal access to mental health care even for those people located in remote areas [39].

For inpatient care, distance decay effects seem to be much less pronounced than for outpatient clinics. In catchment areas with good public transportation systems the home-to-hospital distance might be even completely irrelevant for access to inpatient services. Thus, there seems to be much less empirical basis to decentralize mental hospitals to the same extent as outpatient clinics. However, even if the role of distance would be completely irrelevant for inpatient service utilization, this is not saying that mental hospitals should be located arbitrary in the service provision area. Instead, for patients’ and relatives’ convenience, mental hospitals should be located in or close to the largest communities where most people (and hence most people in need for inpatient treatment) live. This notion is supported by findings showing higher prevalence rates for mental disorders in urban areas [30–35].

### Limitations

Several limitations of this study have to be addressed. First, the ecological cross sectional design does not allow for causal inferences and bears the risk of ecological fallacy. We do not know to what extent the caseloads of the communities were affected by different prevalence rates of mental disorders or by other community-related ecological variables for which no data was available. As our outpatient clinics were located in the most urban areas of the catchment area, which typically have higher prevalence rates of mental disorders [30–35], our results could simply show some decay in the prevalence of mental disorders from urban to rural areas. However, although decreasing prevalence rates of mental disorders or non-controllable ecological variables of the communities might have contributed to the observed distance decay effects, it is rather unlikely that they changed to the same degree with increasing distance from service facilities as the caseloads did.

Second, travel times between patients’ homes and service facilities were calculated using the main public transportation station in each community as starting point. The analyzed travel times were only an approximation of the real travel times of the individuals. However, even if individual door-to-door travel times would have been available from timetables for every inhabitant in the service area, such figures would not have been completely exact (traveling the same way twice almost never takes exactly the same of amount time, e.g. due to delays in public transportation).

Third, our analyses were restricted to those patients who were using the secondary mental health services of the PDAG. While the hospital of the PDAG provides the vast majority of inpatient treatments in the canton of Aargau [24, 25], data on outpatient visits at general practitioners and psychiatrists in private practice was not available to this study. Nevertheless, it is unlikely that the distance decay effects in our outpatient clinics can be completely explained by the omission of outpatient cases and visits at physicians in private practice who run their medical practices in remote areas. Like our outpatient clinics, most private practices are located in urban communities of our service area suggesting that people in remote areas really are at risk of being insufficiently supplied with mental health care.

Fourth, as is usual in mental health services research, diagnoses were not assessed with structured clinical interviews such as the SCID-I [40] and SCIDII [41]. Their application would have been far too time-consuming for a routine clinical care setting. Concerning the reliability of our clinical diagnoses, however, miss-codings might be rare as we only analyzed broad diagnostic categories (ICD-10: F0, F1, F2 etc.). This was confirmed in a recent study in our mental hospital which showed good overall agreement of the commonly used clinical examination technique with SCID I assessments regarding primary diagnoses at the level of these broad diagnostic categories [42].

Fifth, analyses on diagnostic subgroups were restricted to those primary diagnoses which were present in at least *n* = 200 cases, and the 95 (1.7%) cases with missing data on primary diagnosis were excluded from these subgroup analyses. A minimal subsample size of *n* = 200 cases might be considered arbitrary but it was intended to render reliable estimates of the distance decay effects within the most prevalent diagnostic subgroups.

## Conclusions

Our study demonstrated that travel time by public transportation to the closest mental health service facility negatively predicts the utilization of outpatient services for all prevalent mental disorders, even if available ecological (e.g. socioeconomic) characteristics of the communities were controlled for. No such distance decay effect was found for the utilization of inpatient wards, except for organic mental disorders. Based on these findings, outpatient clinics should be most effectively located decentralized and in the largest communities to meet the needs of the population as close as possible to where people live and to avoid remote areas being insufficiently supplied with mental health care. For mental hospitals decentralized location seems to be less important but they should be located in the largest communities of the service provision area.

## Abbreviations

ICD-10: International Statistical Classification of Diseases and Related Health Problems. 10^th^ Revision
PDAG: Psychiatric Services Aargau
SCID-I: Structured Clinical Interview for DSM-IV Axis I Disorders
SCID-II: Structured Clinical Interview for DSM-IV Axis II Personality Disorders
SPSS: PASW Statistics for Windows
